# Gastric bacterial Flora in patients Harbouring *Helicobacter pylori* with or without chronic dyspepsia: analysis with matrix-assisted laser desorption ionization time-of-flight mass spectroscopy

**DOI:** 10.1186/s12876-018-0744-8

**Published:** 2018-01-26

**Authors:** Verima Pereira, Philip Abraham, Sivaramaiah Nallapeta, Anjali Shetty

**Affiliations:** 1 Division of Gastroenterology, P D Hinduja Hospital, V S Marg, Mahim, Mumbai, 400016 India; 2 Bruker Daltonics, Bangalore, India; 3 Division of Microbiology, P D Hinduja Hospital, Mumbai, India

**Keywords:** Gastric microbiome, Gastric microbiota, *Helicobacter pylori* pathogenicity, MALDI-TOF

## Abstract

**Background:**

The gastric microbiota has recently been implicated in the causation of organic/structural gastroduodenal diseases (gastric and duodenal ulcers, gastric cancer) in patients with *Helicobacter pylori (H. pylori)* infection. We aimed to ascertain, in patients harbouring *H. pylori*, the role of the gastric microbiota in the causation of symptoms (chronic dyspepsia) in the absence of organic disease.

**Methods:**

Seventy-four gastric biopsy samples obtained at endoscopy from patients with (*n* = 21) or without (*n* = 53) chronic dyspepsia, and that tested positive by the bedside rapid urease test for *H. pylori* infection, were cultured for detection of *H. pylori* and non-*H. pylori* organisms. The cultured organisms were identified by matrix-assisted laser desorption ionization time-of-flight mass spectroscopy (MALDI-TOF MS).

**Results:**

A total of 106 non-*H. pylori* isolates were obtained from 74 patients’ samples. This included 33 isolates (median 2, range 1–2 per patient) from dyspeptic and 73 (median 2, range 1–2 per patient) from non-dyspeptic patients. These were identified from the Bruker Biotyper 2 database as Staphylococcus spp., Streptococcus spp., Lactobacillus spp., Micrococcus spp., Enterococcus spp., Pseudomonas spp., Escherichia spp., Klebsiella spp. and Bacillus spp., Staphylococcus and Lactobacillus were identified significantly more commonly in dyspeptics and Streptococcus, Pseudomonas, *Escherichia coli* and *Klebsiella pneumoniae* in non-dyspeptics. All identified organisms belonged to the phyla Firmicutes and Proteobacteria.

**Conclusions:**

There is a qualitative difference in the gastric microbial spectrum between patients harbouring *H. pylori* with and without chronic dyspepsia. Whether these organisms have an independent role in the development or prevention of dyspepsia or act in concurrence with *H. pylori* needs study.

## Background

Traditionally, the human stomach has been viewed as an inhospitable environment for microorganisms mainly because of its acidic lumen [1]. With the discovery of *Helicobacter pylori (H. pylori)* and other gastric Helicobacters, and subsequent insight into the mechanisms by which these organisms adapt to the gastric environment, the stomach is no longer considered sterile and the adaptative mechanisms of local organisms are becoming clearer [2].

Bacterial counts in the human stomach were traditionally believed to range from 0 to 10^3^ cfu/g [3]; these figures were based on studies with organisms that can be cultured and identified by standard biochemical techniques. More advanced techniques have brought to light one fact: the normal stomach is host to many more organisms than have been identified by standard culture techniques. Studies found colonisation by a complex microbiota belonging mainly to the Proteobacteria, Firmicutes, Actinobacteria and Fusobacterium phyla, which was clearly different from the microbiota described in the mouth and oesophagus [4]. A high prevalence of non-*H. pylori* bacteria has been found, the majority of which were Streptococcus and Staphylococcus [5, 6].

*H. pylori* infects up to 50% of the world’s population [3]. It has been implicated in the causation of various gastroduodenal (duodenal ulcers, MALT lymphoma, gastric cancer) as well as extraintestinal (e.g., refractory anaemia, idiopathic thrombocytopenic purpura) diseases [7]. Why only a small fraction of those infected will progress to disease development has been the subject of extensive investigation. Several pathogenic factors have been identified within the organism that enable colonisation and progression to disease [3]. Recent attention has also focused on host factors [6–8], with increasing interest in the role of the gastric microbiota particularly in the causation of gastric cancer, in the presence of *H. pylori* infection [9–14]. The role of the human gut microbiota in health and disease in general has been reviewed in detail recently [15].

More common than the development of organic disease is the development of symptoms (chronic dyspepsia) in patients with *H. pylori* infection even in the absence of organic disease (so-called functional dyspepsia). Although the role of *H. pylori* infection in functional dyspepsia is still being debated, eradication of the infection with antibacterial therapy has been shown to provide symptom relief more than with placebo [16]. It is not clear whether the relief is due to eradication of this infection or of any concurrent gastric bacterial population [17]. In support of an important role for non-*H. pylori* bacteria in the causation of disease is the finding in a longitudinal study that treatment for *H. pylori* decreased the occurrence of gastric cancer even in those in whom the organism could not be eradicated [18].

A qualitative difference in the gastric microbiota in persons infected with *H. pylori* has been mentioned [8, 9]; which of the two (*H. pylori* and non-*H. pylori* bacteria) influenced the other is not clear. Little is known about the gastric microbiota in patients with *H. pylori* infection with dyspepsia in the absence of organic disease.

Finally, there is information on the gastric microflora in the West [4, 10, 12–14], but reports on the gastric microbiota in developing countries are few [8]. The gastric microbiota of Indian subjects has not been studied by advanced techniques. Why is this important? We expect it to be different in developing countries from what has been reported from the more hygienic environs of the West. Besides, in the context of *H. pylori* infection, it is worth noting that while antibody to this infection is rather widespread in a country like India [19], the incidence of gastric cancer is not as high as would then be expected [20]; the reasons for this are only speculative [21].

Our primary aim was therefore to characterise the culturable gastric microbiota in patients with *H. pylori* infection with or without chronic dyspepsia in the absence of organic disease. Simultaneously we wished to determine if there is any qualitative difference in the Indian gastric microbiota as compared to that described from the West, in the presence of *H. pylori* infection. For these purposes, we used matrix-assisted laser desorption ionization time-of-flight mass spectrometry (MALDI-TOF MS) for bacterial identification [22]. A higher accuracy is obtained with this technique compared with the phenotypic methods reported before [23].

## Methods

### Subjects

During an 11-month period (September 2013 – August 2014), 74 consecutive patients (40 men, 34 women) undergoing upper gastrointestinal endoscopy for indications decided by their treating physician, who had not received any antibiotic for three months prior or acid-suppression therapy for four weeks prior, and who consented to obtaining endoscopic gastric biopsy for the purpose of the study, were enrolled. Patients who had comorbid illness (e.g., renal failure, chronic NSAID use) or obvious organic disease on endoscopy (ulcers, cancer) that can cause dyspepsia were excluded.

With diligent sterile precautions and after flushing the endoscope working channel with sterile normal saline, two sets each of mucosal biopsy samples were taken using single-use forceps, from the gastric antrum and body. One set was placed in a commercial urea-based strip (Halifax Research Laboratory, Kolkata) for the bedside rapid urease test (RUT) for *H. pylori* infection. The second set was transported in ice packs at 4 °C to the culture laboratory within an hour of collection, using sterile 0.9% saline as transport medium. Samples that yielded positive results on RUT were taken for *H. pylori* culture and further examinations. Tissue was not taken for histologic examination.

The institution’s Ethics Committee reviewed and approved the study protocol (reference number 760-PA-13 dated August 22, 2013) and informed written consent was obtained from each patient.

The 74 patients were divided into two groups, namely, those with chronic dyspepsia (greater than six months’ duration) as the indication for endoscopy (dyspeptics; *n* = 21; median age 48, range 21 to 66 years) and those whose indications for endoscopy were other than dyspepsia (non-dyspeptics; *n* = 53; median age 47, range 22 to 79 years). As mentioned earlier, those with organic disease that can cause dyspepsia were excluded. The dyspeptics group thus had functional dyspepsia, as defined by the Rome III criteria [24]. The non-dyspeptic group included patients with the following indications: dysphagia, anaemia suspected to be due to gastrointestinal blood loss, search for primary cancer, and screening for varices in portal hypertension.

### Tissue culture

The selected samples were dispersed using a homogeniser. Each homogenate was inoculated into sterile tryptic soy broth and the samples were plated on non-selective Brucella blood agar and Mueller Hinton agar plates supplemented with 5% human blood, starch and human serum. They were then incubated at 37 °C under microaerophilic, aerobic and anaerobic conditions for 5 to 7 days.

Small translucent colonies were selected for Giemsa’s staining and tested for urease, catalase and oxidase activity. Curved rods resembling *Helicobacter* that were positive for all the three enzyme activity tests were identified as *H. pylori*.

The non*-H. pylori* colonies were subjected to Gram staining and colony characteristics study (size, shape, colour, margins, opacity, elevation), and to MALDI-TOF MS for identification.

### Identification by MALDI-TOF MS

The method we used was similar to that used by Hu et al. [23] Approprimately 5–10 mg of colony was scraped and suspended in 300 μL deionized water in an Eppendorf tube and mixed; 900 μL ethanol was added to it and further mixed. The sample was then centrifuged at 12000 rpm for 2 min. The supernatant was decanted and centrifuged again and the ethanol was pipetted off without disturbing the bacterial pellet, which was then dried for 2–3 min. Fifty μL formic acid (70% in water) was added to the dry pellet and mixed, and 50 μL acetonitrile was then added. After centrifugation again at 12000 rpm for 2 min, 1 μL of the supernatant containing the bacterial extract was transferred on to the 96-well steel plate and dried. One μL of matrix solution containing saturated solution of cyano-4-hydroxycinnamic acid in 50% acetonitrile + 2.5% trifluoroacetic acid was added and allowed to air dry at room temperature.

Measurement was done with Microflex LT mass spectrometer (Bruker Daltonics; Germany) equipped with a 200 Hz smartbeam laser. The parameter settings were as follows: delay 320 ns; ion source (i) 20 kV; ion source (ii) 18.5 kV, lens voltage 8.5 kV; and mass range 2–15 kDa. Each run was validated with an *Escherichia coli (E. coli)* control sample where the presence of 10 specific proteins ensured that the spectrometer was set properly. Raw spectra of the strains were analysed by MALDI Biotyper 2.0 software (Bruker Daltonics; Germany) using the default settings.

A list of peaks up to 100 was generated. The threshold for peak acceptance was a signal-to-noise (S/N) ratio of 3. After alignment, peaks with a mass-to-charge (m/z) ratio difference of less than 200 ppm were considered to be identical. The peak lists generated were used for matches against the reference library, by directly using the integrated pattern-matching algorithm of the software. All parameters were the same regardless of the bacteria analysed [3] and a score was attributed to each identification.

### Statistical analysis

Fisher’s exact test was used to compare the frequency of micro-organisms in dyspeptic patients and non-dyspeptic patients.

## Results

The ATCC culture of *E. coli* (positive control) was correctly identified with a score of > 2. The yields on both plates (Brucella blood agar and Mueller Hinton agar) were identical for the types of organisms, as identified on MALDI-TOF.

From the 74 biopsy samples, 106 isolates were obtained. These included 33 isolates (median 2, range 1 to 2 per patient) from dyspeptic and 73 (median 2, range 1–2 per patient) from non-dyspeptic patients; there was no difference in the isolation rates between the two groups.

**Fig. 1:**
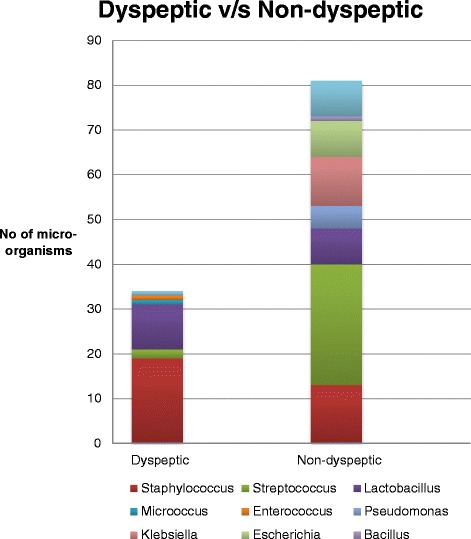
Genera isolated in the two groups (dyspeptics and non-dyspeptics) with *H. pylori* infection

The 106 isolates included 32 of Staphylococcus spp., 29 of Streptococcus spp., 18 of Lactobacillus spp., 11 of *Klebsiella pneumoniae,* 8 of *Escherichia coli,* 5 of *Pseudomonas mosselii*, and one each of Micrococcus, *Enterococcus faecium* and Bacillus (Fig. 1). All the organisms identified belonged to the phyla Firmicutes and Proteobacteria. The Staphylococcus species identified included *Staph. aureus, Staph. equorum, Staph. haemolyticus*, *Staph. auricularis, Staph. hominis, Staph. warneri* and *Staph. xylosus.* The Streptococcus species identified included *Strep. salivarius, Strep. oralis, Strep. sanguinis, Strep. gordonii* and *Strep. parasanguinis.* The Lactobacillus species included *L. brevis* and *L. paracasei. Micrococcus luteus, Enterococcus faecium, Pseudomonas mosselii, Escherichia coli, Klebsiella pneumonia,* and Bacillus spp. were the others identified.

Staphylococcus spp. and Lactobacillus spp. were significantly more commonly identified in dyspeptics; Streptococcus spp., *Pseudomonas mosselii*, *Escherichia coli* and *Klebsiella pneumoniae* were more common in non-dyspeptics (Tables 1 and 2).

**Table 1:** Genera isolated in the two groups (dyspeptics and non-dyspeptics) with *H. pylori* infection

Genera	Dyspeptic (*n* = 21)	Non-dyspeptic (*n* = 53)	*p* value
	No. (%)	No. (%)	
Staphylococcus	19 (90.5)	13 (24.5)	0.00
Streptococcus	2 (9.5)	27 (50.9)	0.0011
Lactobacillus	10 (47.6)	8 (15)	0.0060
Micrococcus	1 (4.8)	0 (0)	0.2837
Enterococcus	1 (4.8)	0 (0)	0.2837
Pseudomonas	0 (0)	5 (9.4)	0.0625
Escherichia	0 (0)	8 (15)	0.007
Klebsiella	0 (0)	11 (20.8)	0.0009
Bacillus	0 (0)	1 (1.9)	1
Total No.	33	73	

Total of 106 species were identified from 74 samples

**Table 2:** Species isolated in the two groups (dyspeptics and non-dyspeptics) with *H. pylori* infection

Micro-organisms	Dyspeptic (*n* = 21)	Non-dyspeptic (*n* = 53)
*Staphylococcus aureus*	8 (38.1%)	5 (9.4%)
*Staphylococcus equorum*	1 (4.8%)	0
*Staphylococcus haemolyticus*	3 (14.3%)	2 (3.8%)
*Staphylococcus auricularis*	1 (4.8%)	0
*Staphylococcus hominis*	2 (9.5%)	3 (5.7%)
*Staphylococcus warneri*	4 (19.1%)	0
*Staphylococcus xylosus*	0	3 (5.7%)
*Streptococcus salivarius*	0	11 (20.8%)
*Streptococcus oralis*	1 (4.8%)	4 (7.6%)
*Streptococcus sanguinis*	0	8 (15.1%)
*Streptococcus gordonii*	1 (4.8%)	0
*Streptococcus parasanguinis*	0	4 (7.6%)
*Lactobacillus brevis*	5 (23.8%)	8 (15.1%)
*Lactobacillus paracasei*	5 (23.8%)	0
*Micrococcus luteus*	1 (4.8%)	0
*Enterococcus faecium*	1 (4.8%)	0
*Pseudomonas mosselii*	0	5 (9.4%)
*Escherichia coli*	0	8 (15.1%)
*Klebsiella pneumoniae*	0	11 (20.8%)
*Bacillus spp*	0	1 (1.9%)
Total No.	33	73

**Fig. 2:**
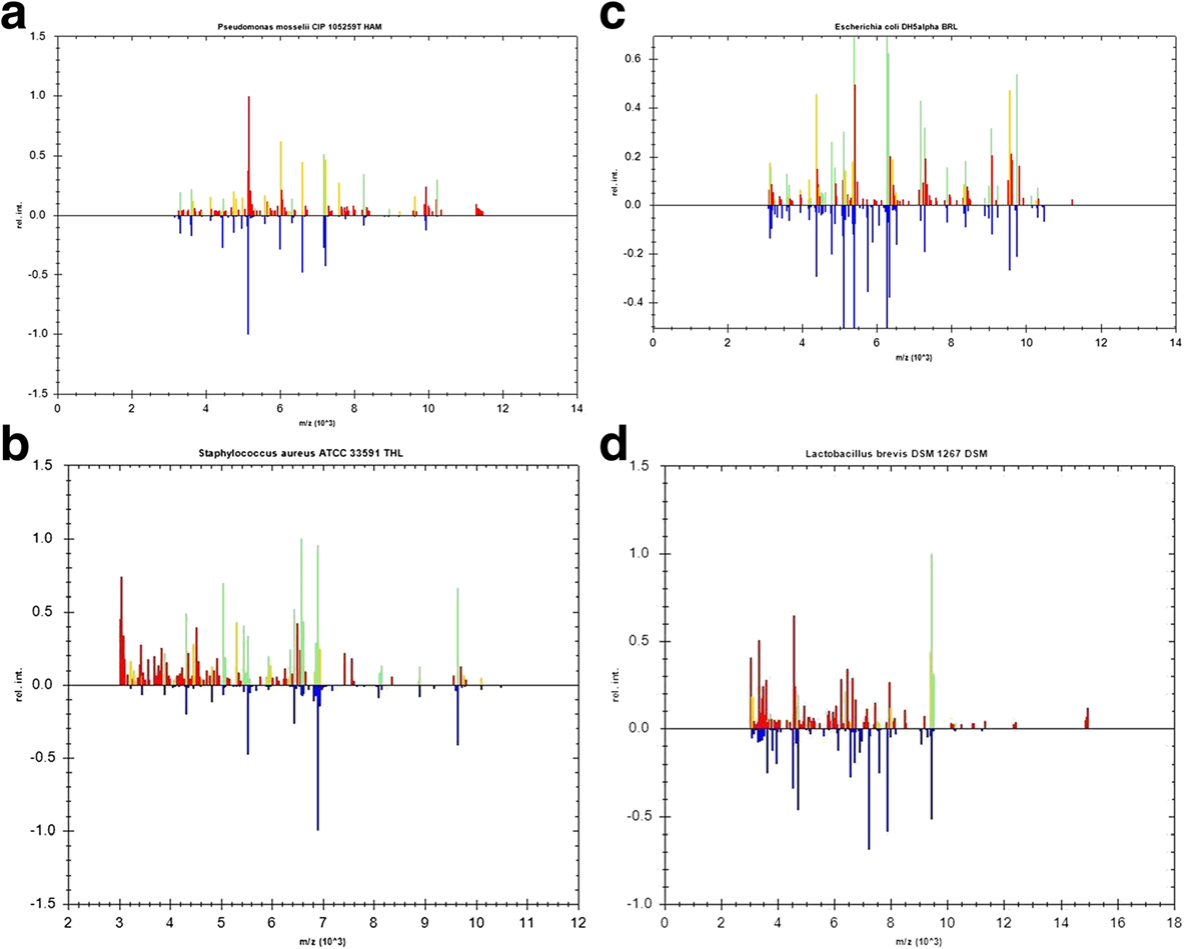
MALDI-TOF MS spectra of 4 commonest organisms isolated (**a** *Pseudomonas mosselii;* **b** *Staphylococcus aureus;* **c** *Escherichia coli;* **d** *Lactobacillus brevis)*

The MALDI-TOF spectra of the four commonest identified species are shown in Fig. 2.

## Discussion

We describe a qualitative difference in the bacterial spectrum in subjects with *H. pylori* infection who do and do not have chronic dyspepsia. The former excluded patients who had obvious organic gastroduodenal or systemic disease that could cause dyspepsia, and met the Rome III criteria for functional dyspepsia [24]. No subgroup analysis (as epigastric pain syndrome and postprandial distress syndrome) was done. We excluded patients who had recently received antibiotic or acid-suppression therapy; none of our patients was on long-term NSAID use. Subsequent to the initiation of our study, the Rome IV diagnostic criteria for functional dyspepsia were introduced [25]. These differ importantly from the Rome III criteria by defining a severity threshold for identifying symptom as ‘bothersome’ and by providing a cut-off for symptom frequency.

Since it would be unethical to perform endoscopies and obtain biopsies in persons without gastroduodenal indications (truly healthy individuals), our comparator group was a diseased-control group that had patients with the infection but without dyspepsia even if they had other structural disease.

Studies from the West have explored the gastric microbiota in the absence and presence of *H. pylori* infection [11]; several studies have also explored the role of the gastric microbiota in the development of structural disease, particularly gastric cancer, in the presence of *H. pylori* infection [10–14]. We attempted to address the issue of why some individuals with *H. pylori* infection develop chronic dyspepsia and others do not, a clinical scenario that is much more common in this infection than the development of structural disease.

We found Staphylococcus spp., Streptococcus spp. and Lactobacillus spp. as the most frequently identified organisms in the samples we studied. The species we isolated on cuture belonged to the phyla Firmicutes and Proteobacteria; Bik et al [4] and others from the West [12–14] reported (on 16 s rRNA sequencing) the presence of Proteobacteria, Firmicutes, Actinobacteria, Bacteroidetes and Fusobacteria phyla in the stomach of those with *H. pylori* infection. Interestingly, the flora in *H. pylori*-positive patients with chronic dyspepsia in our study was dominated by Staphylococcus and Lactobacillus (in that order of prevalence); those without dyspepsia showed dominance by Streptococcus, Staphylococcus and Klebsiella. There was a significant difference in the organisms in the two groups, with Staphylococcus and Lactobacillus more commonly identified in dyspeptics and Streptococcus, Pseudomonas, *Escherichia coli* and *Klebsiella pneumoniae* more common in non-dyspeptics. Lactobacillus is one of the species that has been incriminated in the progression to gastric cancer in *H. pylori*-positive individuals [26].

A role for *H. pylori* in the causation of gastroduodenal disease (acid-peptic diseases, low-grade MALT lymphoma, carcinoma) has been established [27, 28]. However, a question that remains is why only some individuals with this infection develop disease while the majority do not. *H. pylori* virulence factors have been extensively studied in this regard [3]; factors such as the host diet have also been studied, but a clear answer has not been forthcoming. A role for host environment factors (gastric microbiome) has recently received extensive attention [8, 10, 23]. However, we are not aware of studies that attempted to differentiate the gastric microbiota in individuals with *H. pylori* infection, between those with chronic (functional) dyspepsia and those without dyspepsia.

An earlier study by Hu et al [23] had shown that a higher proportion of patients with non-ulcer (functional) dyspepsia had non-*H. pylori* flora as compared to those with gastric ulcer [23], suggesting a role for these concurrent organisms in the causation of symptoms. They, however, did not specify any qualitative difference. They observed an overall high prevalence of Streptococcus, Neisseria, Rothia and Staphylococcus in their patients with *H. pylori* infection; we observed a high prevalence of Staphylococcus spp. and Lactobacillus spp. in chronic dyspeptics and Streptococcus spp., Pseudomonas, *Escherichia coli* and *Klebsiella pneumoniae* in non-dyspeptics. Of these, Staphylococcus and Klebsiella are urease-producing organisms.

We suggest a possibility that some or all of these organisms play a role (bacteria-bacteria, bacteria-host, or bacteria-host-bacteria) in the causation of symptoms in *H. pylori* infection. What the range of interactions among human-associated microbes might be is not clear, nor how this may influence host health or disease [29]. Maldonado-Contreras et al [8] have suggested various mechanisms of interactions between *H. pylori* and non-*H. pylori* organisms in the stomach. The interactions of *H. pylori* with the other bacteria detected in the stomach may be influenced by host response [3, 5, 6, 8, 30–32]. It is likely that *H. pylori* creates special niches that allow the survival and colonisation of bacteria in the stomach [33].

It is well known that colonisation by *H. pylori* leads to changes in the gastric milieu, including raising the pH to > 4, which facilitates colonisation by other bacteria [34]. Multiple non-*H. pylori* organisms have been isolated from the stomach in patients with hypochlorhydria [35]. It could be that the spectrum we obtained was influenced by concomitant *H. pylori* infection. But, if it was, we would expect the influence to be similar in both our study groups, namely, dyspeptics and non-dyspeptics.

On the other hand, Bik et al. [4], who used a metagenomic approach, found no influence of *H. pylori* on the diversity of gastric microbiota. They also showed that there is no difference in the microbiota isolated from the gastric antrum and corpus, which justifies our decision to pool samples from these two regions. The relationship between *H. pylori* and the gastric microbiota is thus still controversial [33].

We also describe, probably for the first time, the culturable gastric flora in an Indian population, as identified using MALDI-TOF MS, a technique that identifies organisms more reliably than the standard culture techniques (morphology and biochemical tests) [36]. The organisms we identified in *H. pylori-*positive individuals may not reflect the spectrum in the general (asymptomatic) Indian population, and so cannot be compared with studies from the healthy population in the West.

Our study had limitations. We used culture (and MALDI-TOF MS) as the identifying technique; this identifies only viable microflora. Metagenomics is a high-end technique that will increase the yield of the microflora by also identifying DNA from dead organisms. Which of these is a more faithful representation of the functional microflora is not clear [26]. Importantly, we did not study the gastric histology in our subjects, and so cannot comment on the presence or absence of inflammation and atrophy, common accompaniments of *H. pylori* infection. This could be a major confounder: the gastric environment, including the pH and the presence of inflammation, can obviously influence the microbial spectrum. Besides, the presence of inflammation and/or atrophy may be an indicator of the virulence of the *H. pylori* strains in these patients, a factor we did not study; the latter could contribute to the development of symptoms in addition to possibly influencing the concomitant microflora. We also had no information on the smoker status of our patients. Finally, we did not explore the potential pathogenicity of bacterial types we isolated. Although some of the organisms we isolated are known to be pathogens in other sites and clinical situations, we do not know yet about their pathogenic ability in the gastric environs.

Information regarding the contribution of the concurrent gastrointestinal microbiome to the development of disease is still in its infancy. Future studies are needed to elucidate whether and to what extent *H. pylori* infection perturbs the established microbiota, and how the concomitant microbiota influence development of symptoms and disease. Such studies should factor in the limitations in our study, in order to get a clearer understanding. Increasing evidence supports the hypothesis that although *H. pylori* is the most relevant, it may not be the only local bacterial culprit leading to gastric diseases. There may be a role for non-*H. pylori* components of the gastric microbiota in both gastric and extragastric diseases. Conversely, some components of the gastric microbiota have been shown to exert antibacterial and probiotic properties, which may be exploited for the prevention and treatment of gastric diseases [37].

## Conclusions

The changes that take place in the gastric environment during *H. pylori* infection are complex and involve several factors. A combination of these would determine not only the composition of the gastric microbiota but also the progress toward different diseases [32]. Our finding of gastric flora dominated by Staphylococcus followed by Lactobacillus in patients with *H. pylori*-positive chronic dyspepsia and by Streptococcus followed by Staphylococcus and Klebsiella (along with gram-negative bacteria) in those without dyspepsia adds an area of interest in the interaction of bacteria in the causation of symptoms. These findings add to the now-popular belief that individual bacteria identified in individual gut disease may not be lone players but may be influenced in their pathogenicity by the community they live in. Although this is an attractive proposition, it is too early to state whether manipulating the concomitant microflora will offer an alternative approach to preventing or managing these diseases or symptoms.
